# Supplementary material for: A universal SARS‐CoV DNA vaccine inducing highly cross‐reactive neutralizing antibodies and T cells
Source: EMBO Mol Med. 2022 Sep 2;14(10):e15821. doi: 10.15252/emmm.202215821 (PMC9538582; doi:10.15252/emmm.202215821)
Supplement: Supplementary file 1 — Expanded View Figures PDF [file EMMM-14-e15821-s004.pdf]

Expanded View Figures

Figure EV1. Sequence alignment and conirmation of protein expression.

A Sequence alignment of the three RBD sequences in the OC-2.4 construct in comparison with the Omicron BA variants.
B Western blot detection of N protein in cell lysates from HEK293 cells 48 h after transfection with indicated plasmid DNA or mRNA. The OC2.4 N protein (70 kDa) is larger than the N protein control (55 kDa) due to the fusion with the M protein.

© 2022 The Authors

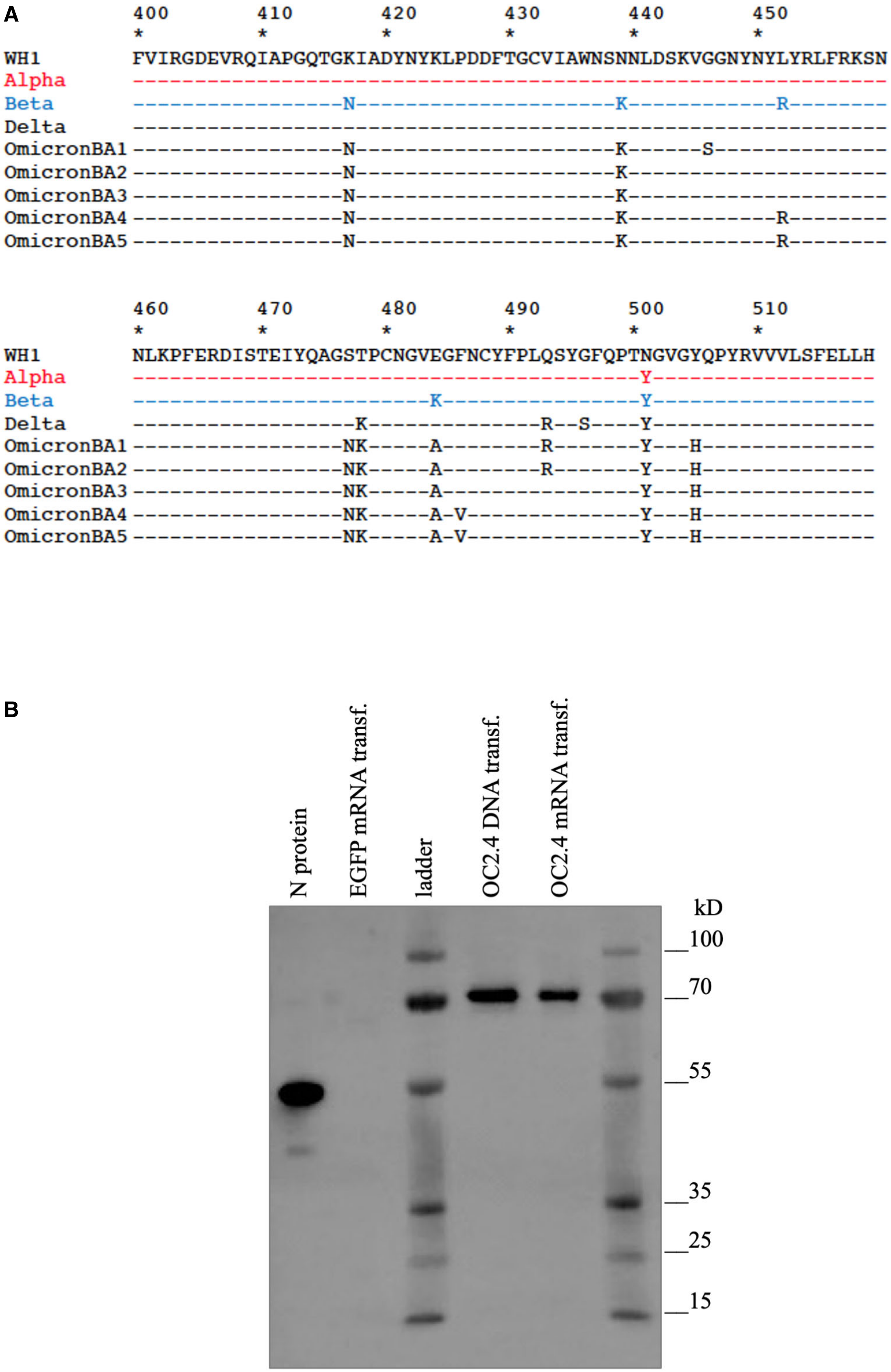
A
400 410 420 430 440 450
WH1 FVIRGDEVRQIAPGQTGKIADYNYKLPDDFTGCVIAWNSNNLDSKVGGNYNYLYRLFRKSN
Alpha ---
Beta -----------------N---------------------K------------R--------
Delta ---
OmicronBA1 -----------------N---------------------K------S--------------
OmicronBA2 -----------------N---------------------K---------------------
OmicronBA3 -----------------N---------------------K---------------------
OmicronBA4 -----------------N---------------------K------------R--------
OmicronBA5 -----------------N---------------------K------------R--------
460 470 480 490 500 510
WH1 NLKPFERDISTEIYQAGSTPCNGVEGFNCYFPLQSYGFQPTNGVGYQPYRVVVLSFELLH
Alpha ---Y------------------
Beta ------------------------K----------------Y------------------
Delta ------------------K--------------R--S----Y------------------
OmicronBA1 -----------------NK-----A--------R-------Y---H--------------
OmicronBA2 -----------------NK-----A--------R-------Y---H--------------
OmicronBA3 -----------------NK-----A----------------Y---H--------------
OmicronBA4 -----------------NK-----A-V--------------Y---H--------------
OmicronBA5 -----------------NK-----A-V--------------Y---H--------------
B
N protein
EGFP mRNA transf.
ladder
OC2.4 DNA transf.
OC2.4 mRNA transf.
kD
100
70
55
35
25
15

Figure EV1.

Figure EV2. Antibodies to the spike (S) and nucleoproteins (N) 2 weeks after the 1st, 2nd, and 3rd dose of OC-2.4.

Data has been given as the raw data in the form of OD at 450 nm at the serial dilution of individual rabbit sera.

© 2022 The Authors

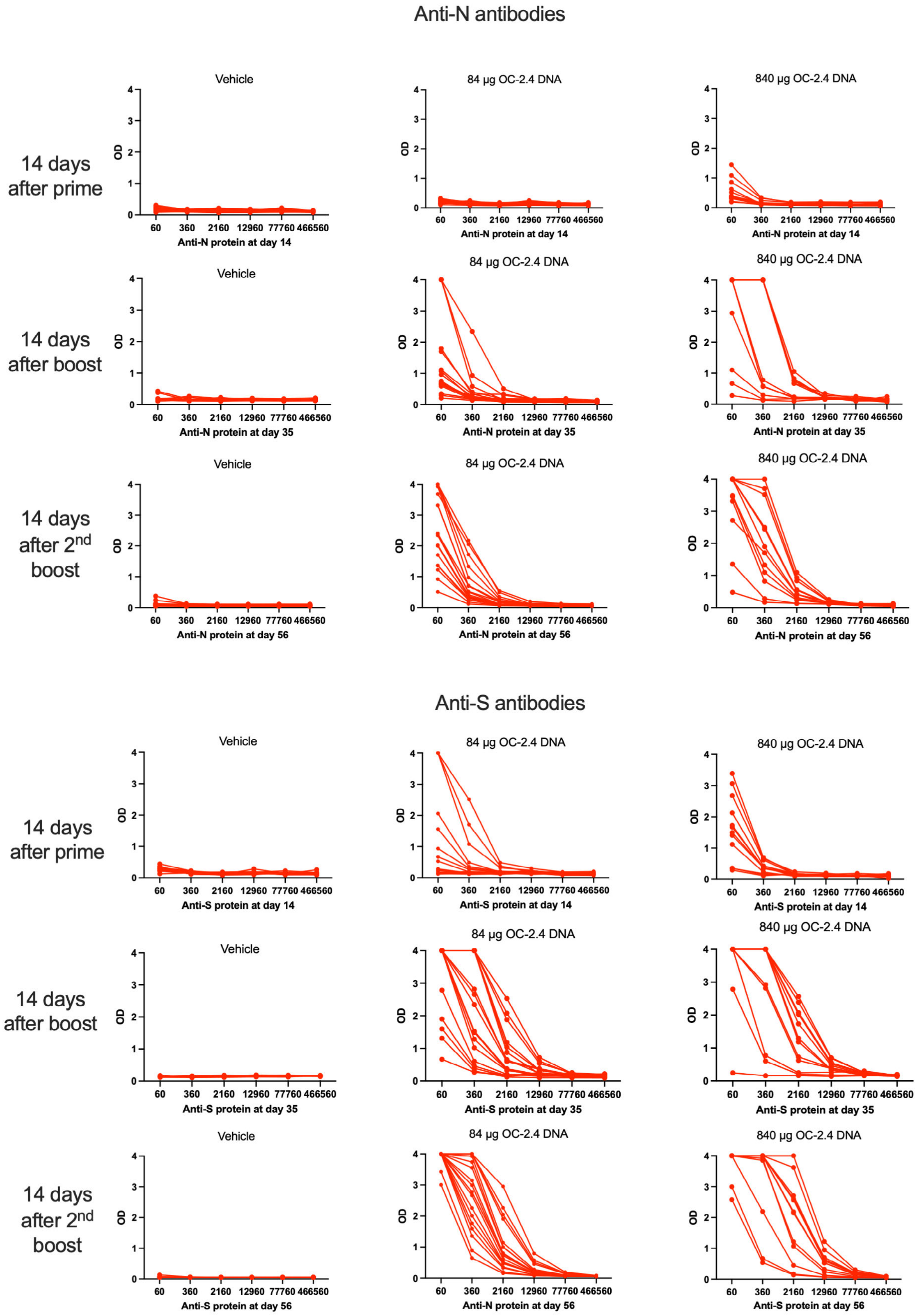
Anti-N antibodies
Vehicle
84 µg OC-2.4 DNA
840 µg OC-2.4 DNA
14 days after prime
14 days after boost
14 days after 2nd boost
OD
60 360 2160 12960 77760 466560
Anti-N protein at day 14
Anti-N protein at day 35
Anti-N protein at day 56
Anti-S antibodies
Anti-S protein at day 14
Anti-S protein at day 35
Anti-S protein at day 56
Anti-N protein at day 56

Figure EV2.

Figure EV3. Immune responses to the N protein by different routes of immunization.

A–E Antibody (A) and T cell (B–E) responses to the N protein in C57BL/6 mice after no immunization (B) or two immunizations 3 weeks apart with recombinant N protein in alumn (rN/Alumn) (C), recombinant N protein in QS21 (rN/QS21) (D), or a DNA plasmid encoding N (WH1) protein (E). Data are given at the number of IFNγ (blue) or IL-2 (red) producing spot forming cells (SFCs)/10^6 splenocytes. The antibody levels were compared between groups using Mann–Whitney U-test (GraphPad Prism) with one asterisk indicating $P < 0.05$ and two asterisks $P < 0.01$.

© 2022 The Authors

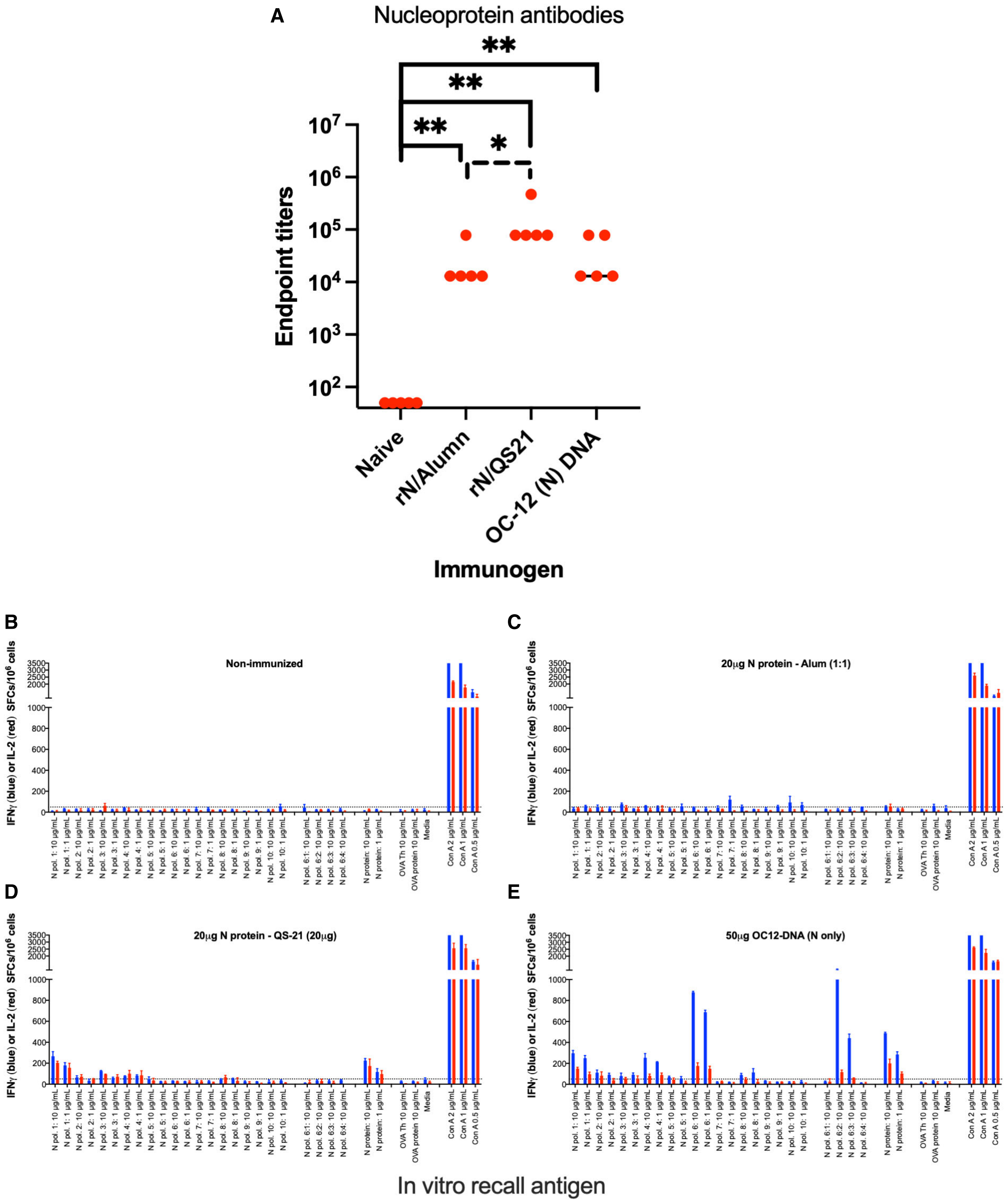
A
Nucleoprotein antibodies
Endpoint titers
Naive
rN/Alumn
rN/QS21
OC-12 (N) DNA
Immunogen
B
Non-immunized
C
20μg N protein - Alum (1:1)
D
20μg N protein - QS-21 (20μg)
E
50μg OC12-DNA (N only)
IFNγ (blue) or IL-2 (red) SFCs/10^6 cells
In vitro recall antigen

Figure EV3.
