# Supplementary material for: A universal SARS‐CoV DNA vaccine inducing highly cross‐reactive neutralizing antibodies and T cells
Source: EMBO Mol Med. 2022 Sep 2;14(10):e15821. doi: 10.15252/emmm.202215821 (PMC9538582; doi:10.15252/emmm.202215821)
Supplement: Supplementary file 2 — Table EV1 [file EMMM-14-e15821-s002.docx]

**EXPANDED VIEW TABLE 1**

Histological findings at the injection site (right quadriceps muscle) in female (A) and male (B) New Zeeland White rabbits at two and 14 days after the fourth injection of vehicle or the indicated dose of DNA using *in vivo* EP.

A)

| **Group no.** | **1** | **2** | **3** | **4** | **5** | **6** |
| --- | --- | --- | --- | --- | --- | --- |
| **Gender** | Female | Female | Female | Female | Female | Female |
| **Treatment dose** | Vehicle (2d) | Vehicle  (14d) | 84 µg DNA  (2d) | 84 µg DNA  (14d) | 840 µg DNA  (2d) | 840 µg DNA  (14d) |
| **No. animals** | 3 | 3 | 3 | 3 | 3 | 3 |
| **INJECTION SITE (m quadriceps)** | 3 | 3 | 2 | 3 | 3 | 3 |
| **Interstitial, inflammatory cell infiltration, multifocal (n)** | 2 | 1 | 2 | 1 | 2 | - |
| **minimal** | - | - | 1 | - | 1 | - |
| **slight** | - | 1 | - | 1 | - | - |
| **moderate** | 1 | - | 1 | - | 1 | - |
| **marked** | 1 | - | - | - | - | - |
| **INJECTION SITE (m quadriceps)** | 3 | 3 | 2 | 3 | 3 | 3 |
| **Degeneration/necrosis, multifocal** | 2 | 1 | 2 | 1 | 1 | - |
| **minimal** | - | - | 1 | - | - | - |
| **slight** | - | - | 1 | 1 | - | - |
| **moderate** | 2 | 1 | - | - | 1 | - |

**Abbreviations and descriptions**

- = no findings, 1 = minimal, 2 = slight, 3 = moderate, 4 = marked, 5 = severe, P = present, M = missing, A = artefact.

**EXPANDED VIEW TABLE 1, continued**

B)

| **Group no.** | **1** | **2** | **3** | **4** | **5** | **6** |
| --- | --- | --- | --- | --- | --- | --- |
| **Gender** | Male | Male | Male | Male | Male | Male |
| **Treatment** | Vehicle (2d) | Vehicle  (14d) | 84 µg DNA  (2d) | 84 µg DNA  (14d) | 840 µg DNA  (2d) | 840µg DNA  (14d) |
| **No. animals** | 3 | 3 | 3 | 3 | 3 | 3 |
| **INJECTION SITE (m quadriceps)** | 3 | 3 | 3 | 3 | 3 | 3 |
| **Interstitial, inflammatory cell infiltration, multifocal** | 2 | 1 | 3 | - | 3 | - |
| **minimal** | - | 1 | - | - | - | - |
| **slight** | - | - | 2 | - | 1 | - |
| **moderate** | 2 | - | - | - | 1 | - |
| **marked** | - | - | 1 | - | 1 | - |
| **INJECTION SITE (m quadriceps)** | 3 | 3 | 3 | 3 | 3 | 3 |
| **Degeneration/necrosis, multifocal** | 2 | 1 | 2 | - | 3 | - |
| **minimal** | - | 1 | 1 | - | - | - |
| **slight** | 1 | - | - | - | 1 | - |
| **moderate** | 1 | - | 1 | - | 2 | - |

**Abbreviations and descriptions**

- = no findings, 1 = minimal, 2 = slight, 3 = moderate, 4 = marked, 5 = severe, P = present, M = missing, A = artefact.
