# Supplementary material for: A universal SARS‐CoV DNA vaccine inducing highly cross‐reactive neutralizing antibodies and T cells
Source: EMBO Mol Med. 2022 Sep 2;14(10):e15821. doi: 10.15252/emmm.202215821 (PMC9538582; doi:10.15252/emmm.202215821)
Supplement: Supplementary file 4 [file EMMM-14-e15821-s006.pdf]

EMBO Molecular Medicine

A universal SARS-CoV DNA vaccine inducing highly crossraective neutralizing antibodies and T cells

Matti Sällberg, Sofia Appelberg, Gustaf Ahlen, Negin Nikouyan, Jingyi Yan, Sofie Weber, Olivia Larsson, Urban Höglund, Soo Aleman, Friedemann Weber, Emma Perlhammre, Johanna Apro, Eva-Karin Gidlund, Ola Tuvesson, Simona Salati, Matteo Cadossi, Hanna tegel, Sophia hober, Lars Frelin, and Ali Mirazimi
DOI: 10.15252/emmm.202215821

Corresponding author: Matti Sällberg (matti.sallberg@ki.se)

Review Timeline:

Submission Date:	4th Feb 22
Editorial Decision:	23rd Feb 22
Revision Received:	13th Jun 22
Editorial Decision:	27th Jun 22
Revision Received:	7th Jul 22
Editorial Decision:	22nd Jul 22
Revision Received:	16th Aug 22
Accepted:	17th Aug 22

Editor: Zeljko Durdevic

Transaction Report:

(Note: With the exception of the correction of typographical or spelling errors that could be a source of ambiguity, letters and reports are not edited. Depending on transfer agreements, referee reports obtained elsewhere may or may not be included in this compilation. Referee reports are anonymous unless the Referee chooses to sign their reports.)

23rd Feb 2022

Dear Dr. Sällberg,

Thank you for the submission of your manuscript to EMBO Molecular Medicine. We have now received feedback from the three reviewers who agreed to evaluate your manuscript. As you will see from the reports below, while the referee #3 is supportive of the study, referees #1 and #2 recognize the interest of the study but also raise serious concerns that should be addressed in a major revision.

Further consideration of a revision that addresses reviewers' concerns in full will entail a second round of review. EMBO Molecular Medicine encourages a single round of revision only and therefore, acceptance or rejection of the manuscript will depend on the completeness of your responses included in the next, final version of the manuscript. For this reason, and to save you from any frustrations in the end, I would strongly advise against returning an incomplete revision.

We would welcome the submission of a revised version within three months for further consideration. Please let us know if you require longer to complete the revision.

Please use this link to login to the manuscript system and submit your revision: Link Not Available

I look forward to receiving your revised manuscript.

Yours sincerely,

Zeljko Durdevic

Zeljko Durdevic
Editor
EMBO Molecular Medicine

***** Reviewer's comments *****

Referee #1 (Remarks for Author):

In their manuscript entitled „Universal SARS-CoV DNA vaccine inducing highly crossrective neutralizing antibodies and T cells" Sofia Appelberg and coworker describe an interesting approach for the development of a more universal Sars-CoV vaccine. Their concept is based on a DNA-vector encoding a fusion protein encompassing the RBD domains of the wt, the beta and alpha variant and the M and N protein. After immunization of mice the authors observe the induction of neutralizing antibodies. In challenge experiments immunized mice were protected from lethal infection.
There are some open points to be addressed.
The authors should show the expression of the OC2.4 construct in transfected cells by immunofluorescence microscopy or Western blot analysis.
The analysis of the T cell response is central for the manuscript. The data should be transferred from the supplement into the manuscript
The structure of the fusion protein is not fully clear. Is the M protein separated by a P2A sequence from the N protein as described in the text?
The titer of S-binding antibodies should be analyzed after the first, second and third vaccination.
If the authors have data describing the Th1 /Th2 polarization these data should be included in the supplement

Referee #2 (Remarks for Author):

Appelberg and colleagues determined the immunogenicity and efficacy of a DNA construct containing receptor binding domain (RBD) loops of the S protein corresponding to the huCoV-19/WH01, Alpha and Beta variants, combined with the M and N proteins of the huCoV-19/WH01 variant to target broadest immune responses. Despite demonstrating some protection against beta virus challenge in mice, there is no evidence that this vaccine is equal or better than licensed vaccines. The main concerns are below:

1. The authors refer to SARS-CoV in the title and text and propose that this approach will give broad reactivity across beta coronaviruses. However, it seems that the vaccine construct contains only genetic elements of SARS-CoV-2, and studies to prove that cross-reactivity or functionality with SARS-CoV or other beta coronaviruses are actually improving the vaccine efficacy are missing. The "SARS-CoV OC-2.4" DNA construct is not clearly described and the author refers to Dai et al Cell 2020 which report a recombinant bivalent RBD approach that does not appear to be relevant for this study.
2. In terms of humoral immunity, the construct does not provide a significant improvement compared to recombinant S protein or spike-DNA.
3. Challenge model with wuhan strain to compare S protein vs DNA is missing, and the challenge model with beta strain does not demonstrate superiority of the DNA construct compared to recombinant S protein. The rationale of use of recombinant S protein + QS21 is not clearly described in the text.
4. T cell cross-reactivity with bat coronavirus is not relevant for the study. Moreover, cross-reactivity levels (figure S3) are below threshold of detection for 4 mice out of 6.
5. Evidence of 60% protection with N protein immunization is not supported histological and RNA evidence, with worsening of clinical score in animals post challenge compared to other treatments. Moreover, this is not relevant for the DNA vaccine.
6. Statistical values, experimental replicates and description of experimental conditions are often missing in the figures and text.
7. There are plenty of typos in the title, abstract, text and figures that makes it difficult to understand the manuscript.

Overall, the manuscript is difficult to read and the conclusions are not supported by relevant experimental evidence. Despite the

aim of expanding breadth of response, additional work is necessary to clarify rationale of the study, to improve the DNA construct and to generate the right controls and the missing data. Thus, I believe the manuscript is not suitable for publication.

Referee #3 (Remarks for Author):

In this elegant study, the group of Matti Sallberg from Sweden describes a universal SARS-CoV DNA vaccine that induces highly cross-reactive neutralizing antibodies and T cells. This group is leading in vaccine development and the data shown convincing. The transgenic moused model used supports the protective role of the induced immune response and also supports a critical role of T cells as has been suggested in the hunan system. Overall, in this reviewers opinion, this is a well performed study that addresses an important and highly relevant topic.

I have only one comment:
The authors use different vaccine strategies, the rationale for which should be discussed or at least be explained.

Replies to comments made by the reviewers.

Referee #1 (Remarks for Author):

In their manuscript entitled „Universal SARS-CoV DNA vaccine inducing highly crossrective neutralizing antibodies and T cells" Sofia Appelberg and coworker describe an interesting approach for the development of a more universal Sars-CoV vaccine. Their concept is based on a DNA-vector encoding a fusion protein encompassing the RBD domains of the wt, the beta and alpha variant and the M and N protein. After immunization of mice the authors observe the induction of neutralizing antibodies. In challenge experiments immunized mice were protected from lethal infection.
There are some open points to be addressed.
*The authors should show the expression of the OC2.4 construct in transfected cells by immunofluorescence microscopy or Western blot analysis.

REPLY: A western blot showing detection of the M-N fusion protein has been enclosed as supplementary figure 1.

*The analysis of the T cell response is central for the manuscript. The data should be transferred from the supplement into the manuscript

REPLY: The T cell data has been included in Figure 2. Also, more data has been included.

*The structure of the fusion protein is not fully clear. Is the M protein separated by a P2A sequence from the N protein as described in the text?

REPLY: The P2A sequence is only present between the RBD and M sequence. The M and N proteins are present as a fusion protein. This has now been clarified in the text, and has been shown in western blot in Supplemental Figure 1.

*The titer of S-binding antibodies should be analyzed after the first, second and third vaccination.

REPLY: This has now been included in Supplemental Figure 2.

*If the authors have data describing the Th1 /Th2 polarization these data should be included in the supplement

REPLY: We have focused on the cross reactivity of the T cells, also the strong IFNγ responses certainly suggest a Th1-like response. This will however be included in the next publication.

Referee #2 (Remarks for Author):

Appelberg and colleagues determined the immunogenicity and efficacy of a DNA construct containing receptor binding domain (RBD) loops of the S protein corresponding to the huCoV-19/WH01, Alpha and Beta variants, combined with the M and N proteins of the huCoV-19/WH01 variant to target broadest immune responses. Despite demonstrating some protection against beta virus challenge in mice, there is no evidence that this vaccine is equal or better than licensed vaccines. The main concerns are below:
1. The authors refer to SARS-CoV in the title and text and propose that this approach will give broad reactivity across beta coronaviruses. However, it seems that the vaccine construct contains only genetic elements of SARS-CoV-2, and studies to prove that cross-reactivity or functionality with SARS-CoV or other beta coronaviruses are actually improving the vaccine efficacy are missing. The "SARS-CoV OC-2.4" DNA construct is not clearly described and the author refers to Dai et al Cell 2020 which report a recombinant bivalent RBD approach that does not appear to be relevant for this study.

REPLY: Thank you for the comment. The description of the SARS-CoV OC-2.4 vaccine has now been better described. We agree that much of the data provided show cross reactivity between SARS-CoV-2 variants. However, we clearly show that vaccine primed T cells are cross reactive with Bat-SARS-CoV. Hence, we feel that it is correct to state that the OC-2.4 vaccine induces immune responses cross reactive with SARS-CoVs.

*2. In terms of humoral immunity, the construct does not provide a significant improvement compared to recombinant S protein or spike-DNA.

REPLY: This is correct. However, the major point of the current vaccine design is to activate both humoral and broadly reactive T cells. Thus, we feel that it is quite impressive that the anti-S responses are comparable to S-based vaccines, but with the addition that new responses to M and N are induced.

*3. Challenge model with wuhan strain to compare S protein vs DNA is missing, and the challenge model with beta strain does not demonstrate superiority of the DNA construct compared to recombinant S protein. The rationale of use of recombinant S protein + QS21 is not clearly described in the text.

REPLY: We feel that the most relevant control is the Beta variant. Also, we agree that the vaccine does not show superiority in the *in vivo* model. However, it is important to stress that we do induce new T cell specificities to highly conserved parts of the virus, such as M and N. This suggests, together with the T cell data, that we have a vaccine that complements the existing vaccines by adding new T cell

repertoires. Importantly, as we show, the activation of N-specific T cells alone has protective properties, supporting the concept.

*4. T cell cross-reactivity with bat coronavirus is not relevant for the study. Moreover, cross-reactivity levels (figure S3) are below threshold of detection for 4 mice out of 6.

REPLY: We disagree with the reviewer's comment. The cross reactivity with Bat N sequences is central to a universal SARS-CoV vaccine, since new viruses are likely to appear from animals such as bats. Thus, even if the S gene is replaced, a prior SARS-CoV infection, or vaccination using our type of vaccine, will still have the chance of offering some degree of protection. Indeed, that is what the experiment with vaccination with N protein alone shows. Thus, the cross reactivity of the vaccine-primed T cells to Bat N sequences is highly relevant.

*5. Evidence of 60% protection with N protein immunization is not supported histological and RNA evidence, with worsening of clinical score in animals post challenge compared to other treatments. Moreover, this is not relevant for the DNA vaccine.

REPLY: We disagree with the reviewer as we feel that the data showing that priming T cells only has a partially protective effect is highly relevant. This is a novel observation that extends data from humans regarding the role of T cells. Although there is histological signs of disease the increased survival clearly shows benefit of priming N specific T cells. Thus, although this was a protein-based vaccination it supports the concept of a protective role of cross-reactive T cells. Importantly, the N protein was from the WH1 strain and protected against a challenge with a Beta strain.

*6. Statistical values, experimental replicates and description of experimental conditions are often missing in the figures and text.

REPLY: We have included statistical analysis when it has been possible to do so. The experimental design has been clarified.

*7. There are plenty of typos in the title, abstract, text and figures that makes it difficult to understand the manuscript.

REPLY: We have corrected the text throughout the manuscript.

Overall, the manuscript is difficult to read and the conclusions are not supported by relevant experimental evidence. Despite the aim of expanding breadth of response, additional work is necessary to clarify rationale of the study, to improve the DNA

construct and to generate the right controls and the missing data. Thus, I believe the manuscript is not suitable for publication.

Referee #3 (Remarks for Author):

In this elegant study, the group of Matti Sallberg from Sweden describes a universal SARS-CoV DNA vaccine that induces highly cross-reactive neutralizing antibodies and T cells. This group is leading in vaccine development and the data shown convincing. The transgenic moused model used supports the protective role of the induced immune response and also supports a critical role of T cells as has been suggested in the human system. Overall, in this reviewers opinion, this is a well performed study that addresses an important and highly relevant topic.

I have only one comment:
The authors use different vaccine strategies, the rationale for which should be discussed or at least be explained.

REPLY: This has now been explained in the text.

27th Jun 2022

Dear Dr. Mirazimi,

Thank you for the submission of your revised manuscript to EMBO Molecular Medicine. We have now heard back from the two referees who we asked to re-evaluate your manuscript. As you will see from the reports below, while the referee #1 supports publication of your manuscript, referee #2 acknowledges the improvements of the revised manuscript but also raises a number of concerns that should be addressed in an additional and final round of major revision. Only one point considering a control group immunized with N protein + QS21 requires additional experimentation, while all the other points should be addressed by additional clarifications and discussion.

Further consideration of a revision that addresses reviewer's concerns in full will entail an additional round of review. Acceptance or rejection of the manuscript will depend on the completeness of your responses included in the next, final version of the manuscript. For this reason, and to save you from any frustrations in the end, I would strongly advise against returning an incomplete revision.

We would welcome the submission of a revised version within three months for further consideration. Please let us know if you require longer to complete the revision.

Please use this link to login to the manuscript system and submit your revision: Link Not Available

I look forward to receiving your revised manuscript.

Yours sincerely,

Zeljko Durdevic

Zeljko Durdevic
Editor
EMBO Molecular Medicine

***** Reviewer's comments *****

Referee #1 (Comments on Novelty/Model System for Author):

The authors adequately addressed all points raised in the previous review.

Referee #1 (Remarks for Author):

The authors adequately addressed all points raised in the previous review. This is a very relevant, well designed and well performed study.

Referee #2 (Comments on Novelty/Model System for Author):

Please see suggested experiments and manuscript review in the comments to the authors.

Referee #2 (Remarks for Author):

The authors focus their discussion on the benefit of inducing N specific T cell response which are cross-reactive with other coronaviruses. When recombinant N protein + adjuvant is used, it results in 60% protection against challenge (Figure 3C). The DNA vaccine is reported to induce both N and spike immunity (Figure 2B,F,G), broadening the immune response, however no added protection is observed in the challenge model (recombinant Wh01 spike protein and DNA vaccine confer full protection against beta challenge, Figure 3C). The study has several limitations which the authors have highlighted in the new version and the flow is clearer. However, I believe the significance of this is limited, and it could be considered for publication provided additional comments are addressed.

Additional comments:

• Clarify why QS21 adjuvant was used
• Remove statements related N specific responses whose data is not shown
• Regarding Figure 2C, additional M and N specific T cell are induced after booster however no significance analysis is given, this should be stated in the text.
• Figure 2E-H should be clearly described in the results section, clarifying that specific IFNy responses are below limit of detection for several mice/group.
• A control group in Figure 2 immunized with N protein + QS21 is missing, this is necessary to define the level of N specific immunity that correlates with in vivo protection, and to compare with DNA-induced immunity
• Clarify in figure le legend and materials&methods what is 1-10 in x axis of Figure 2A-D
• Add p values asterisks in figure 3D

Replies to comments made by the reviewers.

Referee #1 (Comments on Novelty/Model System for Author):

The authors adequately addressed all points raised in the previous review.

Referee #1 (Remarks for Author):

The authors adequately addressed all points raised in the previous review. This is a very relevant, well designed and well performed study.

We thank the reviewer for the comment.

Referee #2 (Comments on Novelty/Model System for Author):

Please see suggested experiments and manuscript review in the comments to the authors.

Referee #2 (Remarks for Author):

The authors focus their discussion on the benefit of inducing N specific T cell response which are cross-reactive with other coronaviruses. When recombinant N protein + adjuvant is used, it results in 60% protection against challenge (Figure 3C). The DNA vaccine is reported to induce both N and spike immunity (Figure 2B,F,G), broadening the immune response, however no added protection is observed in the challenge model (recombinant Wh01 spike protein and DNA vaccine confer full protection against beta challenge, Figure 3C). The study has several limitations which the authors have highlighted in the new version and the flow is clearer. However, I believe the significance of this is limited, and it could be considered for publication provided additional comments are addressed.

Additional comments:
• Clarify why QS21 adjuvant was used

REPLY: The use of the QS21 adjuvant was based on the fact that it is commercially available for research and clinical use. Also, new Expanded View Figure 3 shows that QS21 is superior to alumn, the today most widely used vaccine adjuvant in humans.

• Remove statements related N specific responses whose data is not shown

REPLY: We have now included data that support the statement regarding N antibodies on line 10, page 5.

• Regarding Figure 2C, additional M and N specific T cell are induced after booster however no significance analysis is given, this should be stated in the text.

REPLY: This has now been included in the text. Also, statistical comparisons have been included for both mice and rabbit T cell responses.

• Figure 2E-H should be clearly described in the results section, clarifying that specific IFNy responses are below limit of detection for several mice/group.

REPLY: This has now been clarified in the text that some rabbits failed to develop any T cell responses at any time point.

• A control group in Figure 2 immunized with N protein + QS21 is missing, this is necessary to define the level of N specific immunity that correlates with in vivo protection, and to compare with DNA-induced immunity

REPLY: An additional experiment has been added to clarify this issue, Extended View 3. Also described in Results on page 10.

• Clarify in figure le legend and materials&methods what is 1-10 in x axis of Figure 2A-D

REPLY: Thank you for noticing this. This has now been added in the legend, that this corresponds to peptide pools.

• Add p values asterisks in figure 3D

REPLY: This has now been done.

22nd Jul 2022

Dear Prof. Mirazimi,

Thank you for the submission of your revised manuscript to EMBO Molecular Medicine. I am pleased to inform you that we will be able to accept your manuscript pending the following final amendments:

1) Authorship: In our submission system Ali Mirazimi and Matti Sällberg are indicated as corresponding authors, while in the manuscript only Matti Sällberg is the corresponding author. Please clarify and correct either in the submission system or in the manuscript.
2) E-mail addresses: Please update e-mail addresses for Negin Nikoyan, Johanna Apro, Emma Perlhammre, Ola Tuveson, Olivia Larsson.
3) In the main manuscript file, please do the following:
- Correct/answer the track changes suggested by our data editors by working from the attached/uploaded document.
- Add up to 5 keywords.
- Remove font colour.
- Remove data not shown (p.6).
- Add callouts for the figures 1H and 2G-H.
- Move M&M section after Discussion.
- In M&M, please specify the biosafety level for the experiments with SARS-CoV-2 by adding and amending the following sentence: All experiments with SARS-CoV-2 were performed in a ... level laboratory and with approval from...
- In M&M, statistical paragraph should reflect all information that you have filled in the Authors Checklist, especially regarding randomization, blinding, replication.
- Data availability statement should contain information about data that cannot be published in the manuscript itself (e.g. structural data, high-throughput sequencing or data from large-scale gene expression experiments). If no data are deposited in public repositories, please add the sentence: "This study includes no data deposited in external repositories".

Please check "Author Guidelines" for more information.
https://www.embopress.org/page/journal/17574684/authorguide#availabilityofpublishedmaterial
- Add legend for the figure 2H.
- Please rename "Conflict of Interest" to "Disclosure Statement & Competing Interests". We updated our journal's competing interests policy in January 2022 and request authors to consider both actual and perceived competing interests. Please review the policy https://www.embopress.org/competing-interests and update your competing interests if necessary.
- Correct the reference citation in the text and reference list. In the text, a reference should be cited by author and year of publication. Include a space between a word and the opening parenthesis of the reference that follows. In the reference list, citations should be listed in alphabetical order. Where there are more than 10 authors on a paper, 10 will be listed, followed by "et al.". Please check "Author Guidelines" for more information.
https://www.embopress.org/page/journal/17574684/authorguide#referencesformat
4) Supplementary table: Please upload the table as .xlsx or .doc file and rename it EV Table 1. Also, update the callouts in the main text.
5) Author Contributions: CRediT has replaced the traditional author contributions section because it offers a systematic machine readable author contributions format that allows for more effective research assessment. You are encouraged to use the free text boxes beneath each contributing author's name to add specific details on the author's contribution. More information is available in our guide to authors.
6) Authors Checklist: Please upload the .xlsx file.
7) Synopsis:
- Synopsis image: Please provide a visual abstract as a high-resolution .jpeg file 550 px-wide x (250-400)-px high.
- Synopsis text: Besides the short standfirst, please include 2-5 one sentence bullet points that summarise the paper as a .doc file. Please write the bullet points to summarise the key NEW findings. They should be designed to be complementary to the abstract - i.e. not repeat the same text. We encourage inclusion of key acronyms and quantitative information (maximum of 30 words / bullet point). Please use the passive voice.
- Please check your synopsis text and image before submission with your revised manuscript. Please be aware that in the proof stage minor corrections only are allowed (e.g., typos).
8) For more information: There is space at the end of each article to list relevant web links for further consultation by our readers. Could you identify some relevant ones and provide such information as well? Some examples are patient associations, relevant databases, OMIM/proteins/genes links, author's websites, etc...
9) Press release: Please inform us as soon as possible and latest at the time of submission of the revised manuscript if you plan a press release for your article so that our publisher could coordinate publication accordingly.
10) Please be aware that we use a unique publishing workflow for COVID-19 papers: a non-typeset PDF of the accepted manuscript is published as "Just Accepted" on our website. With respect to a possible press release, we have the option to not post the "Just Accepted" version if you prefer to wait with the press release for the typeset version. Please let us know whether you agree to publication of a "Just accepted" version or you prefer to wait for the typeset version.

11) As part of the EMBO Publications transparent editorial process initiative (see our Editorial at http://embomolmed.embopress.org/content/2/9/329), EMBO Molecular Medicine will publish online a Review Process File (RPF) to accompany accepted manuscripts. This file will be published in conjunction with your paper and will include the anonymous referee reports, your point-by-point response and all pertinent correspondence relating to the manuscript. Let us know whether you agree with the publication of the RPF and as here, if you want to remove or not any figures from it prior to publication. Please note that the Authors checklist will be published at the end of the RPF.

12) Please provide a point-by-point letter INCLUDING my comments as well as the reviewer's reports and your detailed responses (as Word file).

I look forward to reading a new revised version of your manuscript as soon as possible.

Yours sincerely,

Zeljko Durdevic

Zeljko Durdevic
Editor
EMBO Molecular Medicine

***** Reviewer's comments *****

Referee #2 (Remarks for Author):

The authors addressed most of the concerns on the manuscript adding supplementary experimental data and adding more details in the main text. I am ok for publication.

The authors performed the requested editorial changes.

We are pleased to inform you that your manuscript is accepted for publication and is now being sent to our publisher to be included in the next available issue of EMBO Molecular Medicine.

EMBO Press Author Checklist

Matti Sallberg
EMBO Molecular Medicine
EMM-2022-15821

USEFUL LINKS FOR COMPLETING THIS FORM
The EMBO Journal - Author Guidelines
EMBO Reports - Author Guidelines
Molecular Systems Biology - Author Guidelines
EMBO Molecular Medicine - Author Guidelines

Reporting Checklist for Life Science Articles (updated January 2022)
This checklist is adapted from Materials Design Analysis Reporting (MDAR) Checklist for Authors. MDAR establishes a minimum set of requirements in transparent reporting in the life sciences (see Statement of Task: 10.31222/osf.io/9sm4x). Please follow the journal's guidelines in preparing your manuscript.
Please note that a copy of this checklist will be published alongside your article.

Abridged guidelines for figures
1. Data
The data shown in figures should satisfy the following conditions:
- the data were obtained and processed according to the field's best practice and are presented to reflect the results of the experiments in an accurate and unbiased manner.
- ideally, figure panels should include only measurements that are directly comparable to each other and obtained with the same assay.
- plots include clearly labeled error bars for independent experiments and sample sizes. Unless justified, error bars should not be shown for technical replicates.
- if n<5, the individual data points from each experiment should be plotted. Any statistical test employed should be justified.
- Source Data should be included to report the data underlying figures according to the guidelines set out in the authorship guidelines on Data Presentation.

2. Captions
Each figure caption should contain the following information, for each panel where they are relevant:
- a specification of the experimental system investigated (eg cell line, species name).
- the assay(s) and method(s) used to carry out the reported observations and measurements.
- an explicit mention of the biological and chemical entity(ies) that are being measured.
- an explicit mention of the biological and chemical entity(ies) that are altered/varied/perturbed in a controlled manner.
- the exact sample size (n) for each experimental group/condition, given as a number, not a range;
- a description of the sample collection allowing the reader to understand whether the samples represent technical or biological replicates (including how many animals, litters, cultures, etc.).
- a statement of how many times the experiment shown was independently replicated in the laboratory.
- definitions of statistical methods and measures:
  - common tests, such as t-test (please specify whether paired vs. unpaired), simple χ2 tests, Wilcoxon and Mann-Whitney tests, can be unambiguously identified by name only, but more complex techniques should be described in the methods section;
  - are tests one-sided or two-sided?
  - are there adjustments for multiple comparisons?
  - exact statistical test results, e.g., P values = x but not P values < x;
  - definition of 'center values' as median or average;
  - definition of error bars as s.d. or s.e.m.

Please complete ALL of the questions below.
Select "Not Applicable" only when the requested information is not relevant for your study.

Materials

Newly Created Materials	Information included in the manuscript?	In which section is the information available? (Reagents and Tools Table, Materials and Methods, Figures, Data Availability Section)
New materials and reagents need to be available; do any restrictions apply?	Yes	Materials And Methods

Antibodies	Information included in the manuscript?	In which section is the information available? (Reagents and Tools Table, Materials and Methods, Figures, Data Availability Section)
For **antibodies** provide the following information:<br>- Commercial antibodies: RRID (if possible) or supplier name, catalogue number and or/clone number<br>- Non-commercial: RRID or citation	Yes	Materials And Methods

DNA and RNA sequences	Information included in the manuscript?	In which section is the information available? (Reagents and Tools Table, Materials and Methods, Figures, Data Availability Section)
**Short novel DNA or RNA including primers, probes**: provide the sequences.	Yes	Materials And Methods

Cell materials	Information included in the manuscript?	In which section is the information available? (Reagents and Tools Table, Materials and Methods, Figures, Data Availability Section)
**Cell lines:** Provide species information, strain. Provide accession number in repository **OR** supplier name, catalog number, clone number, and/**OR** RRID.	Yes	Materials And Methods
**Primary cultures:** Provide species, strain, sex of origin, genetic modification status.	Not Applicable	
Report if the cell lines were recently **authenticated** (e.g., by STR profiling) and tested for mycoplasma contamination.	Not Applicable	

Experimental animals	Information included in the manuscript?	In which section is the information available? (Reagents and Tools Table, Materials and Methods, Figures, Data Availability Section)
**Laboratory animals or Model organisms:** Provide species, strain, sex, age, genetic modification status. Provide accession number in repository **OR** supplier name, catalog number, clone number, **OR** RRID.	Yes	Materials And Methods
**Animal observed in or captured from the field:** Provide species, sex, and age where possible.	Not Applicable	
Please detail **housing and husbandry conditions**.	Yes	Materials And Methods

Plants and microbes	Information included in the manuscript?	In which section is the information available? (Reagents and Tools Table, Materials and Methods, Figures, Data Availability Section)
**Plants:** provide species and strain, ecotype and cultivar where relevant, unique accession number if available, and source (including location for collected wild specimens).	Not Applicable	
**Microbes:** provide species and strain, unique accession number if available, and source.	Yes	Materials And Methods

Human research participants	Information included in the manuscript?	In which section is the information available? (Reagents and Tools Table, Materials and Methods, Figures, Data Availability Section)
If collected and within the bounds of privacy constraints report on age, sex and gender or ethnicity for all study participants.	Not Applicable	

Core facilities	Information included in the manuscript?	In which section is the information available? (Reagents and Tools Table, Materials and Methods, Figures, Data Availability Section)
If your work benefited from core facilities, was their service mentioned in the acknowledgments section?	Not Applicable	

Design

Study protocol	Information included in the manuscript?	In which section is the information available? (Reagents and Tools Table, Materials and Methods, Figures, Data Availability Section)
If study protocol has been **pre-registered, provide DOI in the manuscript**. For clinical trials, provide the trial registration number **OR** cite DOI.	Not Applicable	
Report the **clinical trial registration number** (at ClinicalTrials.gov or equivalent), where applicable.	Not Applicable	

Laboratory protocol	Information included in the manuscript?	In which section is the information available? (Reagents and Tools Table, Materials and Methods, Figures, Data Availability Section)
Provide DOI OR other citation details if **external detailed step-by-step protocols** are available.	Yes	Materials And Methods

Experimental study design and statistics	Information included in the manuscript?	In which section is the information available? (Reagents and Tools Table, Materials and Methods, Figures, Data Availability Section)
Include a statement about **sample size** estimate even if no statistical methods were used.	Yes	Materials And Methods
Were any steps taken to minimize the effects of subjective bias when allocating animals/samples to treatment (e.g. **randomization procedure**)? If yes, have they been described?	Not Applicable	
Include a statement about **blinding** even if no blinding was done.	Yes	Materials And Methods
Describe **inclusion/exclusion criteria** if samples or animals were excluded from the analysis. Were the criteria pre-established?<br><br>If sample or data points were omitted from analysis, report if this was due to attrition or intentional exclusion and provide justification.	Not Applicable	No samples excluded
For every figure, are **statistical tests** justified as appropriate? Do the data meet the assumptions of the tests (e.g., normal distribution)? Describe any methods used to assess it. Is there an estimate of variation within each group of data? Is the variance similar between the groups that are being statistically compared?	Yes	Materials And Methods

Sample definition and in-laboratory replication	Information included in the manuscript?	In which section is the information available? (Reagents and Tools Table, Materials and Methods, Figures, Data Availability Section)
In the figure legends: state number of times the experiment was **replicated** in laboratory.	Yes	Materials And Methods
In the figure legends: define whether data describe **technical or biological replicates**.	Yes	Materials And Methods

Ethics

Ethics	Information included in the manuscript?	In which section is the information available? (Reagents and Tools Table, Materials and Methods, Figures, Data Availability Section)
Studies involving **human participants**: State details of **authority granting ethics approval** (IRB or equivalent committee(s), provide reference number for approval.	Not Applicable	
Studies involving **human participants**: Include a statement confirming that **informed consent** was obtained from all subjects and that the experiments conformed to the principles set out in the WMA Declaration of Helsinki and the Department of Health and Human Services Belmont Report.	Not Applicable	
Studies involving **human participants:** For publication of **patient photos**, include a statement confirming that consent to publish was obtained.	Not Applicable	
Studies involving experimental **animals**: State details of **authority granting ethics approva**l (IRB or equivalent committee(s), provide reference number for approval. Include a statement of compliance with ethical regulations.	Yes	Materials And Methods
Studies involving **specimen and field samples:** State if relevant **permits** obtained, provide details of authority approving study; if none were required, explain why.	Not Applicable	

Dual Use Research of Concern (DURC)	Information included in the manuscript?	In which section is the information available? (Reagents and Tools Table, Materials and Methods, Figures, Data Availability Section)
Could your study fall under dual use research restrictions? Please check biosecurity documents and list of **select agents and toxins** (CDC): https://www.selectagents.gov/sat/list.htm	Yes	Materials And Methods
If you used a select agent, is the security level of the lab appropriate and reported in the manuscript?	Yes	Materials And Methods
If a study is subject to dual use research of concern regulations, is the name of the **authority granting approval and reference number** for the regulatory approval provided in the manuscript?	Not Applicable	

Reporting

The MDAR framework recommends adoption of discipline-specific guidelines, established and endorsed through community initiatives. Journals have their own policy about requiring specific guidelines and recommendations to complement MDAR.

Adherence to community standards	Information included in the manuscript?	In which section is the information available? (Reagents and Tools Table, Materials and Methods, Figures, Data Availability Section)
State if relevant guidelines or checklists (e.g., **ICMJE, MIBBI, ARRIVE, PRISMA**) have been followed or provided.	Not Applicable	
For **tumor marker prognostic studies,** we recommend that you follow the **REMARK** reporting guidelines (see link list at top right). See author guidelines, under 'Reporting Guidelines'. Please confirm you have followed these guidelines.	Not Applicable	
For **phase II and III randomized controlled trials**, please refer to the **CONSORT** flow diagram (see link list at top right) and submit the CONSORT checklist (see link list at top right) with your submission. See author guidelines, under 'Reporting Guidelines'. Please confirm you have submitted this list.	Not Applicable	

Data Availability

Data availability	Information included in the manuscript?	In which section is the information available? (Reagents and Tools Table, Materials and Methods, Figures, Data Availability Section)
Have **primary datasets** been deposited according to the journal's guidelines (see 'Data Deposition' section) and the respective accession numbers provided in the Data Availability Section?	Yes	Materials And Methods
Were **human clinical and genomic datasets** deposited in a public access-controlled repository in accordance to ethical obligations to the patients and to the applicable consent agreement?	Not Applicable	
Are **computational models** that are central and integral to a study available without restrictions in a machine-readable form? Were the relevant accession numbers or links provided?	Not Applicable	
If publicly available data were reused, provide the respective **data citations in the reference list**.	Not Applicable	
